# Genetic Environment Surrounding *bla*_OXA-55-like_ in Clinical Isolates of Shewanella algae Clade and Enhanced Expression of *bla*_OXA-55-like_ in a Carbapenem-Resistant Isolate

**DOI:** 10.1128/mSphere.00593-21

**Published:** 2021-10-13

**Authors:** Yuki Ohama, Kotaro Aoki, Sohei Harada, Tatsuya Nagasawa, Tomoo Sawabe, Lisa Nonaka, Kyoji Moriya, Yoshikazu Ishii, Kazuhiro Tateda

**Affiliations:** a Department of Microbiology and Infectious Diseases, Toho University Graduate School of Medicine, Tokyo, Japan; b Department of Infection Control and Prevention, The University of Tokyo Hospital, Tokyo, Japan; c Department of Microbiology and Infectious Diseases, Toho University School of Medicine, Tokyo, Japan; d Laboratory of Microbiology, Faculty of Fisheries Sciences, Hokkaido University, Hakodate, Hokkaido, Japan; e Department of Nutritional Science, Faculty of Human Life Science, Shokei Gakuen, Kumamoto, Japan; Antimicrobial Development Specialists, LLC

**Keywords:** *Shewanella algae*, *Shewanella chilikensis*, *Shewanella carassii*, *bla*_OXA-55_, carbapenem-hydrolyzing class D β-lactamases, beta-lactamases, carbapenem

## Abstract

Although *Shewanella* spp. are most frequently isolated from marine environments; more rarely, they have been implicated in human infections. *Shewanella* spp. are also recognized as the origin of genes for carbapenem-hydrolyzing class D β-lactamases. Due to the spread globally among *Enterobacterales* in recent years, risk assessments of both clinical and environmental *Shewanella* strains are urgently needed. In this study, we analyzed the whole-genome sequences of 10 clinical isolates and 13 environmental isolates of *Shewanella* spp. and compared them with those of *Shewanella* species strains registered in public databases. In addition, the levels of *bla*_OXA-55-like_ transcription and β-lactamase activity of a carbapenem-resistant Shewanella algae isolate were compared with those of carbapenem-susceptible S. algae clade isolates. All clinical isolates were genetically identified as S. algae clade (S. algae, Shewanella chilikensis, and Shewanella carassii), whereas all but one of the environmental isolates were identified as various *Shewanella* spp. outside the S. algae clade. Although all isolates of the S. algae clade commonly possessed an approximately 12,500-bp genetic region harboring *bla*_OXA-55-like_, genetic structures outside this region were different among species. Among S. algae clade isolates, only one showed carbapenem resistance, and this isolate showed a high level of *bla*_OXA-55-like_ transcription and β-lactamase activity. Although this study documented the importance of the S. algae clade in human infections and the relationship between enhanced production of OXA-55-like and resistance to carbapenems in S. algae, further studies are needed to elucidate the generalizability of these findings.

**IMPORTANCE** *Shewanella* spp., which are known to carry chromosomally located *bla*_OXA_ genes, have mainly been isolated from marine environments; however, they can also cause infections in humans. In this study, we compared the molecular characteristics of clinical isolates of *Shewanella* spp. with those originating from environmental sources. All 10 clinical isolates were genetically identified as members of the Shewanella algae clade (S. algae, *S. chilikensis*, and *S. carassii*); however, all but one of the 13 environmental isolates were identified as *Shewanella* species members outside the S. algae clade. Although all the S. algae clade isolates possessed an approximately 12,500-bp genetic region harboring *bla*_OXA-55-like_, only one isolate showed carbapenem resistance. The carbapenem-resistant isolate showed a high level of *bla*_OXA-55-like_ transcription and β-lactamase activity compared with the carbapenem-susceptible isolates. To confirm the clinical significance and antimicrobial resistance mechanisms of the S. algae clade members, analysis involving more clinical isolates should be performed in the future.

## INTRODUCTION

*Shewanella* spp. are oxidase-positive, catalase-positive, nonfermentative Gram-negative motile bacilli characterized by the production of hydrogen sulfide (1). *Shewanella* spp. are most frequently detected in marine environments but has also been isolated from extreme environments, such as polar (Shewanella livingstonensis and Shewanella frigidmarina) and high-pressure (Shewanella banthica and Shewanella violacea) environments (2). *Shewanella* spp. have also been implicated in human infections, but only infrequently. *Shewanella* spp. are susceptible to most antimicrobial agents, including β-lactams, but several clinical isolates resistant to carbapenems have been reported (3, 4).

Although only five species of *Shewanella* were recognized in 1998, this number had increased to 57 by 2011. Selection of an appropriate analytical method is known to be crucial for the accurate species identification of *Shewanella* isolates, and 16S rRNA and *gyrB* sequencing, which are common genetic analysis methods for species identification, do not have sufficient resolution to distinguish between *Shewanella* species (1). Therefore, the names of identified species reported in the academic literature and public databases are often unintentionally incorrect. In microbial identification in the clinical microbiology laboratory, distinguishing between the two clinically important species Shewanella algae and Shewanella putrefaciens is possible by examining whether they can grow in Salmonella-*Shigella* agar at 4 or 42°C or in the presence of 6.5% NaCl (1). However, reports show that it is difficult to accurately distinguish these species using the automated identification instruments commonly used in microbiology laboratories (3).

Since the early 2000s, *Shewanella* spp. have been acknowledged to possess genes for carbapenem-hydrolyzing class D β-lactamases. Based on the analysis of a limited number of strains, Shewanella oneidensis and S. algae have been shown to respectively carry *bla*_OXA-54_ and *bla*_OXA-55_ in their genomes (5, 6). The recent spread of the *bla*_OXA-48_ group among *Enterobacterales* prompted the search for the origin of these genes, and Shewanella xiamenensis has been identified as the progenitor (7, 8). The gene structures in the vicinity of *bla*_OXA_ have been shown to be similar among a variety of *Shewanella* species, with a gene encoding “Peptidase_C15” protein upstream and a *lysR* gene downstream, but there is a scarcity of information on the broader genetic environment (8, 9).

Although recent genetic analysis studies have confirmed the presence of *bla*_OXA-55_ in S. algae isolates, the extent of the involvement of *bla*_OXA-55_ in β-lactam resistance, especially carbapenem resistance, in this species is unknown (4). In addition, whether the genetic environment of *bla*_OXA_ is common to the different strains of S. algae and to closely related species of the S. algae clade, such as Shewanella carassii, Shewanella chilikensis, and Shewanella indica, is unconfirmed.

In this study, we analyzed whole-genome sequences of the collected clinical and environmental strains of *Shewanella* spp. and compared them with the genomes of *Shewanella* species type strains and other strains registered in GenBank. In particular, we genetically characterized the S. algae clade, which is of great clinical importance, by examining *bla*_OXA-55-like_ and the genetic environment surrounding the gene, and we examined the relationship between the susceptibility of *Shewanella* spp. to β-lactams, including carbapenems, the levels of *bla*_OXA-55-like_ transcription and OXA-55-like expression, and the amino acid sequences of the expressed proteins.

## RESULTS

### Species identification of study isolates by ANI of genome sequence.

The whole-genome sequencing data obtained in this study and registered in GenBank were clustered based on an average nucleotide identity (ANI) of ≥95%, and the bacterial species were identified according to the presence of type strains in each cluster (Table 1; see Table S1 in the supplemental material). Among the 22 isolates sequenced in this study, 14 formed a cluster with the type strains, and their bacterial species were determined. In addition, four isolates were type strains, and the remaining four isolates were either independent or clustered with other study isolates only: hence, the species could not be determined. Nine isolates were identified as S. algae, and all except one were clinical isolates, including ATCC 49138, a clinical isolate registered as *S. haliotis* by ATCC. In addition, two isolates respectively identified as *S. carassii* and *S. chilikensis* belonging to the S. algae clade were also clinically isolated. Taken together, all clinical isolates analyzed were identified as S. algae clade members, and all but one of the environmental isolates in this study were identified as *Shewanella* spp. outside the S. algae clade.

**TABLE 1:** Bacterial isolates investigated in this study

Strain name	Type strain	Provider	Isolation date	Source	Analyzed in this study				
					Species	*bla*_OXA_ (% nucleotide identity)^*a*^	Other resistance gene(s) (% nucleotide identity)^*b*^	Accession no.	Reference
TUM4442	No	Toho University Omori Medical Center	August 1998	Clinical isolate (blood)	Shewanella algae	*bla*_OXA-SHE_ (99.2)	*qnrA7* (99.1)	AP024610.1	14
TUM17377	No	The University of Tokyo Hospital	January 2012	Clinical isolate (blood)	Shewanella chilikensis	*bla*_OXA-SHE_ (95.9)	*qnrA2* (100)	AP024611.0	This study
TUM17378	No	The University of Tokyo Hospital	June 2014	Clinical isolate (biopsy specimen of soft tissue)	Shewanella algae	*bla*_OXA-SHE_ (99.2)	*qnrA7* (99.2)	AP024612.1	This study
TUM17379	No	The University of Tokyo Hospital	September 2014	Clinical isolate (drainage fluid from abdominal cavity)	Shewanella algae	*bla*_OXA-SHE_ (99.0)	*qnrA3* (99.9)	AP024613.1	This study
TUM17382	No	The University of Tokyo Hospital	July 2015	Clinical isolate (bile)	Shewanella algae	*bla*_OXA-SHE_ (99.0)	*qnrA3* (99.4)*qnrA5* (99.4)	AP024614.1	This study
TUM17383	No	The University of Tokyo Hospital	July 2015	Clinical isolate (drainage fluid from abdominal cavity)	Shewanella algae	*bla*_OXA-SHE_ (99.0)	*qnrA3* (99.4)	AP024615.1	This study
TUM17384	No	The University of Tokyo Hospital	July 2015	Clinical isolate (drained abscess from extremities)	Shewanella algae	*bla*_OXA-SHE_ (99.0)	*qnrA3* (99.4)	AP024616.1	This study
TUM17386	No	The University of Tokyo Hospital	September 2015	Clinical isolate (stool)	Shewanella algae	*bla*_OXA-SHE_ (99.2)	*qnrA3* (99.7)	AP024617.1	This study
TUM17387	No	The University of Tokyo Hospital	August 2016	Clinical isolate (biopsy specimen of intestinal mucosa)	Shewanella carassii	*bla*_OXA-SHE_ (95.7)	*qnrA1* (96.0)	AP024618.1	This study
ATCC 49138	No	National Collection of Type Cultures	Unknown	Clinical isolate	Shewanella algae	*bla*_OXA-55_ (99.7)	*aadA7* (100)*qnrA3* (99.2)*sul1* (100)*qacE* (100)	AP024609.1	This study
JCM11561	Yes	National Research Institute of Fisheries Science	June 1998	Environmental isolate (black porgy intestine, Hiroshima, Japan)	Shewanella schlegeliana	None	None	BPEX00000000.1	22
JCM11563	Yes	National Research Institute of Fisheries Science	June 1995	Environmental isolate (saury intestine, Pacific Ocean off Japan)	Shewanella sairae	None	None	BPEY00000000.1	22
JCM21037 (=ATCC 51192)	Yes	Central Fisheries Research Institute	Unknown	Environmental isolate (red alga)	Shewanella algae	*bla*_OXA-SHE_ (99.3)	*qnrA3* (99.9)	JAAXPX000000000.1	This study
LMG23746	Yes	Central Fisheries Research Institute	1995-2001	Environmental isolate (marine fish in the Baltic Sea)	Shewanella algidipiscicola	None	None	BPFB00000000.1	23
T147	Yes	Central Fisheries Research Institute	1995-2001	Environmental isolate (marine fish in the Baltic Sea)	Shewanella glacialipiscicola	*bla*_OXA-548_ (81.2)	None	BPFC00000000.1	23
ATCC BAA-1207	Yes	Central Fisheries Research Institute	September 2001	Environmental isolate (marine fish)	Shewanella hafniensis	*bla*_OXA-548_ (98.3)	None	BPFD00000000.1	This study
ATCC BAA-642	No	Central Fisheries Research Institute	1997	Environmental isolate (marine invertebrate)	Shewanella colwelliana	None	None	BPFF00000000.1	This study
ATCC BAA-1206	No	Central Fisheries Research Institute	September 2001	Environmental isolate (marine fish)	Shewanella morhuae	*bla*_OXA-551_ (82.0)	None	BPFE00000000.1	This study
KT0246	No	Hokkaido University	April 2007	Environmental isolate (Apostichopus japonicus small intestine, Ainuma, Japan)	*Shewanella* sp. (unidentified)	None	None	BPEV00000000.1	24
c952	No	Hokkaido University	April 2007	Environmental isolate (Apostichopus japonicus small intestine, Ainuma, Japan)	*Shewanella* sp. (unidentified)	None	None	BPEW00000000.1	25
MBTL60-007	No	Ehime University	April 2004	Environmental isolate (sediment at aquaculture site along the coast of Seto Inland Sea)	*Shewanella* sp. (unidentified)	None	*aadA2b* (99.9)*sul2* (100)*sul1* (100)*tet*(M) (99.8)*tet*(C) (99.7)*tet*(B) (99.9)*qacE* (100)*mph*(G) (99.9)*mef*(C) (100)*mph*(F) (100)	BPET00000000.1	26
MBTL60-112-B1	No	Ehime University	May 2004	Environmental isolate (sediment at aquaculture site along coast of Seto Inland Sea)	*Shewanella* sp. (unidentified)	None	None	BPEZ00000000.1	26
MBTL60-112-B2	No	Ehime University	May 2004	Environmental isolate (sediment at aquaculture site along coast of Seto Inland Sea)	*Shewanella* sp. (unidentified)	None	None	BPFA00000000.1	26
MBTL60-118	No	Ehime University	May 2004	Environmental isolate (sediment at aquaculture site along coast of Seto Inland Sea)	Shewanella colwelliana	None	*tet*(B) (99.8)	BPEU00000000.1	26

a The *bla*_OXA_ gene identified with ResFinder and the sequence similarity between the *bla*_OXA_ of each strain and the reference genes (e.g., *bla*_OXA-SHE_, *bla*_OXA-55_, etc.) are shown. Although *bla*_OXA-55_ and *bla*_OXA-SHE_ were separately registered as reference genes of ResFinder, nucleotide sequences of these genes have 99.0% similarity, and both were regarded as *bla*_OXA-55-like_ in this study.

b Percentages indicate the nucleotide sequence similarity of each detected resistance gene to the reference genes in the ResFinder database.

10.1128/mSphere.00593-21.1 TABLE S1 Whole-genome sequencing data of *Shewanella* spp. collected from GenBank and used in this study. Shown are the species and strain name in GenBank, the accession number, and the species identified by average nucleotide identity (ANI) in this study. Download Table S1, XLSX file, 0.1 MB. Copyright © 2021 Ohama et al. 2021 Ohama et al. https://creativecommons.org/licenses/by/4.0/ This content is distributed under the terms of the Creative Commons Attribution 4.0 International license.

### Core genome SNP-based phylogenetic analysis of S. algae clade isolates.

**FIG 1:**
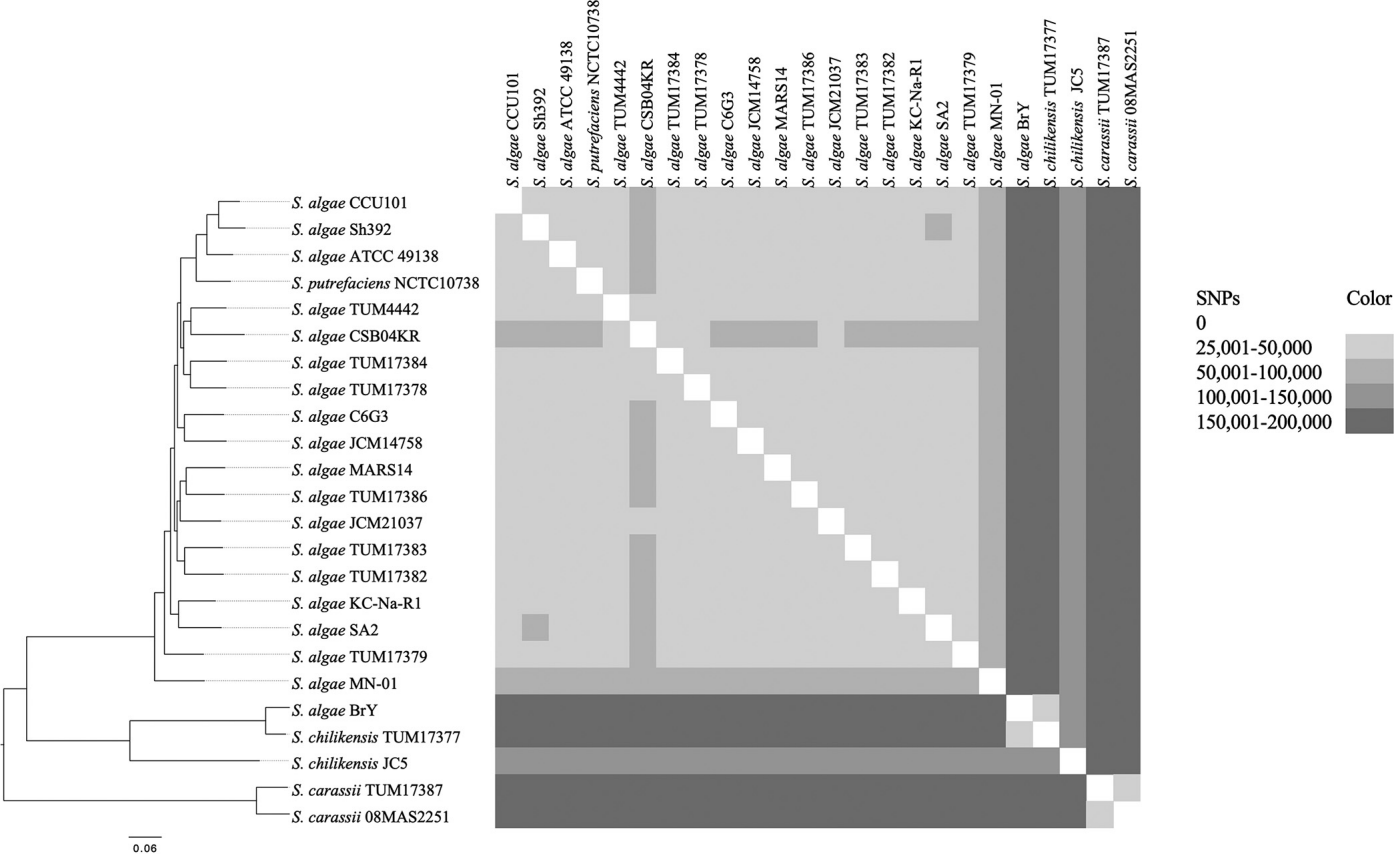
Clonal relatedness of Shewanella algae clade isolates. The core genome size was 60.6% (2,977,854/4,909,921 bp) of the reference genome in the alignment, S. algae TUM17379 (accession no. AP024613.1). The numbers of SNPs are shown in the heat map. BrY was identified as *S. chilikensis* using the average nucleotide identity (Table S1).

The core genome of the S. algae clade, which covered 60.6% (2,977,854 bp) of the genome of the reference isolate, was used for the single-nucleotide polymorphism (SNP)-based phylogenetic analysis. Isolates of the same species clustered into the same branch of the phylogenetic tree (Fig. 1). SNP differences ranged from 26,452 bp to 173,920 bp (median, 49,369 bp).

### Comparison of *bla*_OXA_ and the surrounding genetic environment.

**FIG 2:**
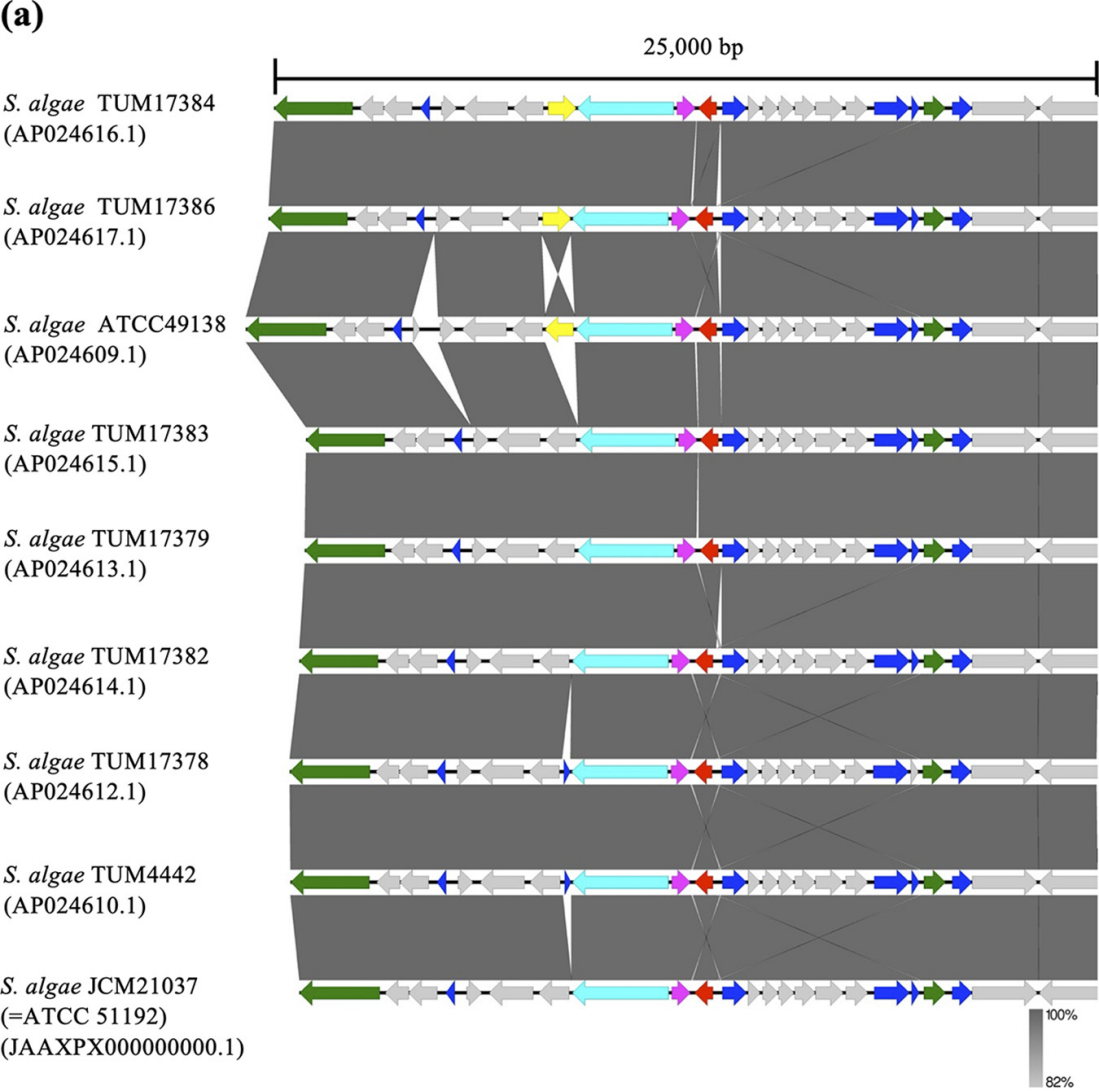

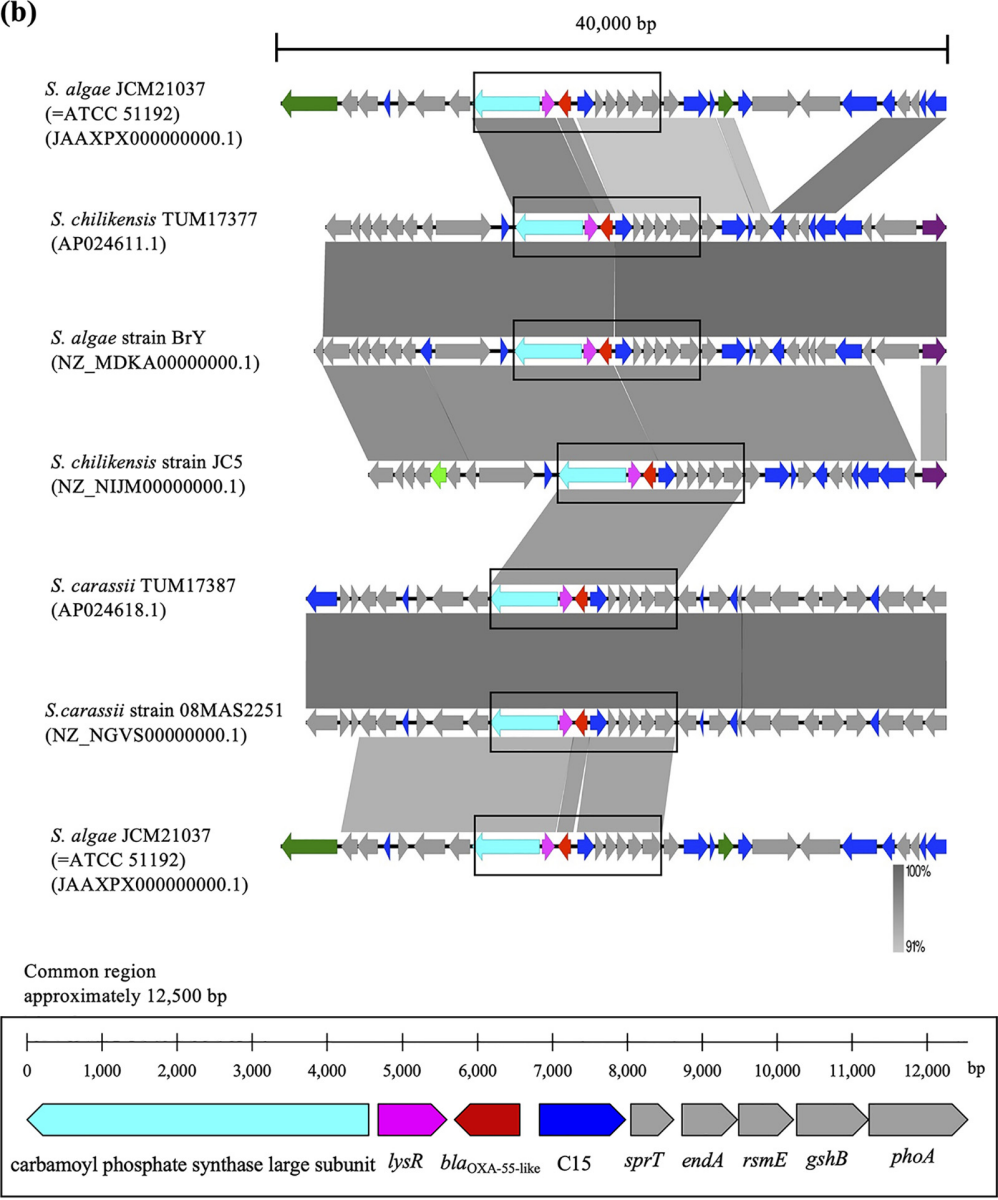
Comparison of the genetic environments of *bla*_OXA-55-like_ in (a) Shewanella algae and (b) Shewanella chilikensis and Shewanella carassii isolates. Shaded areas between nucleotide sequences indicate regions of high similarity. Block arrows indicate confirmed or putative open reading frames (ORFs) and their orientations. Arrow size is proportional to the predicted ORF length. The common genetic region among three species is enclosed in a square and presented in enlarged form below using *S. chilikensis* strain JC5 as the representative. The color code is as follows: magenta, LysR family transcriptional regulator; red, *bla*_OXA-55-like_; green, protease; blue, hypothetical protein; purple, membrane protein; lime, transporter; cyan, carbamoyl phosphate synthase large subunit; yellow, IS*4* family transposase; gray, others.

We compared the 25,000-bp genetic regions surrounding *bla*_OXA-55-like_ among the chromosomes of S. algae isolates. The genetic structures around *bla*_OXA-55-like_ were almost identical, except for minor differences, which included the presence of a gene for an IS*4* family transposase in three of the nine isolates (Fig. 2a). The comparison of a 40,000-bp genetic region surrounding *bla*_OXA-55-like_ of S. algae clade isolates, including non-algae species (*S. chilikensis* and *S. carassii*), is shown in Fig. 2b. These three species shared an approximately 12,500-bp common genetic region around *bla*_OXA-55-like_, including the adjacent C15 gene and *lysR*. However, beyond this shared region, the nucleotide sequences were unique to each species.

### Antimicrobial susceptibilities of S. algae clade isolates and E. coli DH5α carrying *bla*_OXA-55-like_ from S. algae isolates.

Among the S. algae clade isolates, only TUM17384 was nonsusceptible to piperacillin and imipenem, while all other isolates were susceptible to piperacillin, cefotaxime, ceftazidime, aztreonam, imipenem, and meropenem (Table 2). TUM17377 (*S. chilikensis*) and TUM17378 (*S. carassii*) had lower MICs for cefazolin compared to S. algae isolates and also showed low MICs for ampicillin and ampicillin/clavulanic acid.

**TABLE 2:** Antimicrobial susceptibility of isolates

Isolate	MIC (μg/ml) of^*a*^:								
	AMP	AMPC	PIP	CFZ	CTX	CAZ	ATM	IPM	MEM
S. algae									
TUM4442	64	64	1	>128	≤0.06	0.25	0.25	1	≤0.06
JCM21037 (=ATCC 51192)	8	8	1	>128	0.25	0.5	0.5	4	0.125
TUM17378	16	16	≤0.06	>128	0.125	0.5	0.5	1	0.125
TUM17379	0.25	≤0.06	0.5	>128	≤0.06	0.25	0.25	1	≤0.06
TUM17382	4	≤0.06	0.5	>128	≤0.06	0.125	0.25	2	≤0.06
TUM17383	16	≤0.06	1	>128	≤0.06	0.25	0.5	4	0.25
TUM17384	>128	>128	>128	>128	0.125	0.5	0.5	8	1
TUM17386	8	0.125	1	>128	≤0.06	0.25	0.5	2	≤0.06
ATCC 49138	0.5	≤0.06	0.5	64	≤0.06	0.125	0.25	0.5	≤0.06
*S. chilikensis* TUM17377	≤0.06	≤0.06	0.125	1	≤0.06	≤0.06	≤0.06	≤0.06	≤0.06
*S. carassii* TUM17387	≤0.06	≤0.06	0.25	8	≤0.06	0.125	0.125	0.125	≤0.06
E. coli									
DH5α(pHSG298-*bla*_OXA_TUM4442_)	16	2	4	8	≤0.06	≤0.06	≤0.06	0.125	≤0.06
DH5α(pHSG298-*bla*_OXA_JCM21037 [=ATCC 51192]_)	16	2	4	2	≤0.06	≤0.06	≤0.06	0.125	≤0.06
DH5α(pHSG298-*bla*_OXA_TUM17378_)	16	2	1	4	≤0.06	≤0.06	≤0.06	0.125	≤0.06
DH5α(pHSG298-*bla*_OXA_TUM17379_)	16	2	2	4	≤0.06	≤0.06	≤0.06	0.125	≤0.06
DH5α(pHSG298-*bla*_OXA_TUM17382_)	16	2	2	2	≤0.06	≤0.06	≤0.06	0.125	≤0.06
DH5α(pHSG298-*bla*_OXA_TUM17383_)	4	1	1	2	≤0.06	≤0.06	≤0.06	0.125	≤0.06
DH5α(pHSG298-*bla*_OXA_TUM17384_)	32	4	8	8	≤0.06	≤0.06	≤0.06	0.125	≤0.06
DH5α(pHSG298-*bla*_OXA_TUM17386_)	16	2	8	4	≤0.06	≤0.06	≤0.06	0.125	≤0.06
DH5α(pHSG298-*bla*_OXA_ATCC49138_)	8	2	2	4	≤0.06	≤0.06	≤0.06	0.125	≤0.06
DH5α(pHSG298)	2	1	1	2	≤0.06	≤0.06	≤0.06	0.125	≤0.06

a AMP, ampicillin; AMPC, ampicillin-clavulanic acid; PIP, piperacillin; CFZ, cefazolin; CTX, cefotaxime; CAZ, ceftazidime; ATM, aztreonam; IPM, imipenem; MEM, meropenem.

While some Escherichia coli DH5α isolates carrying the pHSG298 vector with *bla*_OXA-55-like_ derived from S. algae isolates showed higher MICs for ampicillin, piperacillin, and cefazolin than DH5α isolates carrying pHSG298 without *bla*_OXA-55-like_, no isolates showed increased MICs for cefotaxime, ceftazidime, aztreonam, imipenem, or meropenem (Table 2).

### Comparison of the transcription levels of *bla*_OXA-55-like_.

**FIG 3:**
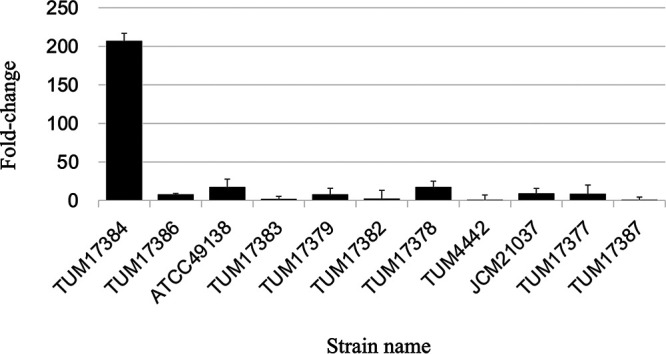
Comparison of *bla*_OXA-55-like_ transcription levels evaluated by RT-PCR. Quantitative RT-PCR was performed on S. algae clade isolates, and the transcript levels of *bla*_OXA-55-like_ in the isolates were compared using the ΔΔ*C_T_* method.

TUM17384 had an approximately 200-fold-higher level of *bla*_OXA-55-like_ transcription than TUM4442 (the reference isolate) (Fig. 3). The remaining isolates had *bla*_OXA-55-like_ transcription levels of 1.8- to 17.5-fold compared with that of TUM4442.

### Comparison of β-lactamase activity in crude enzyme solution.

We measured the β-lactamase activity of the crude enzyme solution of S. algae clade isolates. The hydrolytic activities for nitrocefin, benzylpenicillin, oxacillin, and meropenem of the crude enzyme solution of TUM17384, which had a high initial velocity of *bla*_OXA-55-like_ enzyme, were approximately 55, 4, 26, and 9 times higher, respectively, than the average initial velocity of the remaining 10 isolates (Table 3).

**TABLE 3:** β-Lactamase activity of the crude enzyme solution of S. algae clade isolates

Strain name	Enzyme activity (U/mg)^*a*^				
	Oxacillin	Benzylpenicillin	Nitrocefin	Meropenem	Imipenem
TUM17384	6,541 ± 2,630	2,969 ± 428	1,635 ± 1,397	459 ± 129	166 ± 5
TUM17386	1,079 ± 1,030	508 ± 36	15 ± 2	72 ± 11	15 ± 1
ATCC 49138	233 ± 239	1,916 ± 1,979	3 ± 0	43 ± 5	28 ± 1
TUM17383	260 ± 34	608 ± 442	5 ± 0	60 ± 2	17 ± 1
TUM17379	109 ± 114	545 ± 48	3 ± 1	53 ± 2	13 ± 1
TUM17382	92 ± 102	482 ± 190	37 ± 3	39 ± 5	10 ± 0
TUM17378	86 ± 36	689 ± 166	89 ± 3	53 ± 18	10 ± 1
TUM4442	51 ± 22	639 ± 153	126 ± 137	49 ± 10	16 ± 2
JCM 21037	99 ± 97	462 ± 132	19 ± 2	37 ± 11	12 ± 1
TUM17377	105 ± 40	572 ± 73	3 ± 0	46 ± 14	17 ± 1
TUM17387	390 ± 76	393 ± 82	3 ± 1	43 ± 4	11 ± 1

a The values shown are the mean ± SD from 3 measurements. The initial velocity of the enzyme was determined by measuring the following substrates at 30°C for 1 min under the following respective conditions: nitrocefin (Calbiochem, San Diego, CA) at 482 nm (Δε = +15,000 M^−1 ^cm^−1^), benzylpenicillin (Sigma-Aldrich, St. Louis, MO, USA) at 233 nm (Δε = −775 M^−1 ^cm^−1^), meropenem (Sigma-Aldrich) at 298 nm (Δε = −9,000 M^−1 ^cm^−1^), oxacillin (Sigma-Aldrich) at 260 nm (Δε = +370 M^−1 ^cm^−1^), and imipenem (Banyu Pharmaceutical) at 278 nm (Δε = −6,500 M^−1 ^cm^−1^). The parameters are presented as the average from three independent measurements.

### Alignment of OXA-55-like β-lactamase amino acid.

The amino acid sequences of the OXA-55-like from the 11 S. algae strains, OXA-55, and OXA-SHE were aligned, and one to eight amino acid substitutions were detected (Table 4). The amino acid sequences of OXA-55-like_TUM17378_ and OXA-55-like_TUM17386_ were identical, as were those of OXA-55-like_TUM4442_, OXA-55-like_TUM17379_, and OXA-55-like_TUM17382_ (Table 4).

**TABLE 4:** OXA-55-like β-lactamase amino acid alignment of S. algae clade isolates

β-Lactamase^*a*^	Amino acid position in OXA-SHE^*b*^																	
	4	33	35	38	41	67	98	99	106	128	167	194	198	202	239	261	269	286
OXA-SHE	G	E	T	S	G	S	I	P	E	A	K	V	R	D	R	V	S	Q
OXA-55	G	E	T	S	**C**	S	**L**	P	E	**V**	**E**	V	R	D	R	V	S	Q
OXA-55-like_JCM21037_	G	E	T	**C**	G	S	I	P	E	A	**E**	V	R	D	R	V	S	Q
OXA-55-like_TUM17378_, OXA-55-like_TUM17386_^*c*^	G	E	T	S	G	S	I	P	E	A	**D**	V	R	D	R	V	S	Q
OXA-55-like_TUM4442_, OXA-55-like_TUM17379_, OXA-55-like_TUM17382_^*d*^	G	E	**A**	S	G	S	I	P	E	A	**D**	V	R	D	R	V	S	Q
OXA-55-like_TUM17383_	G	E	T	S	G	S	I	P	E	A	**E**	V	R	D	R	**I**	S	**L**
OXA-55-like_TUM17384_	G	E	T	S	G	S	I	P	E	A	**E**	V	R	D	R	**I**	S	Q
OXA-55-like_ATCC49138_	G	E	T	S	**S**	S	**L**	P	E	**V**	**E**	V	R	D	R	V	S	Q
OXA-55-like_TUM17377_	G	**A**	T	S	G	**C**	I	P	**Q**	A	**Q**	**L**	**Q**	D	**H**	**I**	**G**	Q
OXA-55-like_TUM17387_	**A**	E	T	S	G	**C**	I	**S**	E	A	**Q**	V	**Q**	**G**	R	**I**	S	Q

a The amino acid sequences in OXA-SHE and OXA-55 sequences were converted from nucleotide sequences obtained from GenBank to amino acid sequences. The GenBank accession numbers of *bla*_OXA-SHE_ and *bla*_OXA-SHE_ are AY066004 and AY343493, respectively.

b Of 13 OXA-type β-lactamase alignments, this table shows only the positions where amino acid substitution was detected. The amino acid position was counted from the initiation codon from OXA-SHE, including the estimated signal peptide. Boldface indicates the amino acid is different from OXA-SHE.

c There were two silent mutations on *bla*_OXA-55-like_ between TUM17378 and TUM17386.

d There were two silent mutations on *bla*_OXA-55-like_ between TUM4442 and TUM17379 and between TUM4442 and TUM17382. There were four silent mutations on *bla*_OXA-55-like_ between TUM17379 and TUM17382.

## DISCUSSION

In this study, we analyzed the whole-genome sequences of *Shewanella* isolates recovered from clinical or environmental sources and publicly available *Shewanella* isolates and compared them with genomic data deposited in GenBank. All nine clinical isolates and one publicly available isolate originating from a clinical sample were identified as members of the S. algae clade. Although all isolates in the S. algae clade shared an approximately 12,500-bp genetic region harboring *bla*_OXA-55-like_, the genetic structures outside this region differed among the species. Among the S. algae clade, only one S. algae isolate showed carbapenem resistance, and this strain had high levels of *bla*_OXA-55-like_ transcription and β-lactamase activity.

The difficulties with the correct identification of species of *Shewanella* are universally recognized. All S. algae clade isolates that had been identified in the clinical setting (those isolated at University of Tokyo Hospital [UTH]), were incorrectly identified by automated instruments and matrix-assisted laser desorption ionization–time of flight mass spectrometry (MALDI-TOF MS). In addition, some of the isolates’ draft whole-genome sequences, which were available in GenBank, were registered under the name of different *Shewanella* species. These results are expected, because it is difficult to identify *Shewanella* spp. using automated instruments and 16S rRNA sequencing (1, 3). Although misidentification by MALDI-TOF MS has been reported previously (10), it is assumed that this was partly due to the insufficient registration of reference spectrum data for *Shewanella* spp. in the database. The MALDI Biotyper Library version 9, which was used at UTH, has data for only four strains of S. putrefaciens and one strain each of S. algae, S. baltica, *S. fidelis*, S. frigidimarina, and *S. profunda*. We expect that enrichment of the data will facilitate the correct identification of *Shewanella* spp. with MALDI-TOF MS (11).

All clinical isolates analyzed in this study were identified as S. algae clade members, and all except one of the environmental isolates were identified as various *Shewanella* spp. outside the S. algae clade. Although most of the clinical isolates were from a single hospital, some of the isolates were different species (*S. chilikensis* and *S. carassii*), and 26,452 or more SNPs were found among the S. algae isolates. This suggests each patient acquired the S. algae clade isolates independently rather than by nosocomial transmission. In the past, human *Shewanella* species infections were mainly caused by S. algae and S. putrefaciens; however, assuming they have been correctly identified, most cases in recent years appear to have been caused by S. algae (1, 3). The fact that all of our clinical isolates were S. algae clade supports this assumption.

While isolates of the S. algae clade, other than S. algae itself, have been mainly detected in environmental samples, there are sporadic reports of these being isolated from human specimens, including those suspected to contain causative pathogens of infectious disease (12, 13). It is unclear whether the clinical characteristics of these species differ from those of S. algae due to the limited number of cases reported so far. However, the identification of an *S. chilikensis* isolate from clinical samples in this study suggested the ability of this species to cause human infections. We also verified that *bla*_OXA-55-like_ is shared among the S. algae clade species, but the genetic structure surrounding *bla*_OXA-55-like_ differs among species, except for in the vicinity of *bla*_OXA-55-like_. However, as the genomes of only a limited number of isolates, including those registered in GenBank, have been analyzed, it will be necessary to accumulate more information in the future.

The presence of *bla*_OXA-55-like_ in S. algae has been repeatedly documented by previous studies (5, 6). This study confirmed the universal occurrence of *bla*_OXA-55-like_ in the chromosomes of S. algae isolates, and the diversity of *bla*_OXA-55-like_ nucleotide sequences among the isolates is suggestive of the stable long-term persistence of the gene. Although several carbapenem-resistant isolates of S. algae have been reported, the mechanisms and the extent of involvement of *bla*_OXA-55-like_ in carbapenem resistance are unknown (4, 14). In this study, we cloned *bla*_OXA-55-like_ genes with slightly different nucleotide sequences from nine S. algae isolates and introduced them into E. coli DH5α, but no differences in the MICs of broad-spectrum cephalosporins or carbapenems were observed. Therefore, there was no evidence that the minor differences in the *bla*_OXA-55-like_ possessed by S. algae isolates have a direct effect on their susceptibility to carbapenems. However, the only carbapenem-resistant isolate showed high levels of *bla*_OXA-55-like_ transcription and β-lactamase activity, indicating that the increased production of OXA-55-like contributes to carbapenem resistance via an unknown mechanism.

Imipenem had a higher MIC value than meropenem in the S. algae clade isolates, but the crude enzyme hydrolysis activity of each S. algae clade isolate for imipenem was not significantly different from that for meropenem. The lower affinity of imipenem for PBP2 of the S. algae clade strains compared with meropenem is believed to have influenced the difference in MIC between imipenem and meropenem (Table 3). However, we have not been able to obtain data on the affinity of imipenem for PBP2. Even though the transcript level of *bla*_OXA-55-like_ in strain ATCC 49138 was not high (Fig. 3), the strain’s benzylpenicillin hydrolytic activity was higher than those of the other strains, except TUM17384, but the amino acid sequence alignment of OXA-55-like did not reveal any amino acid substitutions characteristic of OXA-55-like in ATCC 49138 that would explain its hydrolytic activity.

There were several limitations to this study. First, only a small number of clinical isolates were analyzed, and most were collected from a single institution. Although all clinical isolates were S. algae clade members, it was unclear whether this predominance is a general feature of *Shewanella* species isolated in clinical settings. Because only one carbapenem-resistant isolate of S. algae was analyzed, we are unsure whether the high production of OXA-55-like observed in this isolate is a common characteristic of carbapenem-resistant isolates of S. algae. Second, clinical information on the patients was not available, and the detailed characteristics of the *Shewanella* infections could not be analyzed. Third, the mechanism that leads to the high production of OXA-55-like in the carbapenem-resistant S. algae isolate was not investigated. If *bla*_OXA-55-like_ expression is regulated by other genes, differences in the genetic backgrounds of the different S. algae clade species may affect the frequency of carbapenem resistance.

In conclusion, we performed whole-genome sequencing analysis of *Shewanella* spp. detected in clinical and environmental samples and confirmed the dominance of the S. algae clade in the clinical isolates. In addition, we found that S. algae clade strains share an approximately 12,500-bp genetic region that harbors the gene *bla*_OXA-55-like_, but the genetic structures outside this region were different among the different clade species, and the expression of *bla*_OXA-55-like_ was increased only in the carbapenem-resistant isolate. To confirm the clinical significance and antimicrobial resistance mechanisms of S. algae clade members, analysis involving more clinical isolates should be performed in the future.

## MATERIALS AND METHODS

### Bacterial isolates.

Nine isolates of *Shewanella* spp. were detected in different patients at the University of Tokyo Hospital (UTH) between November 2014 and August 2016, seven of which were stored at −80°C and used in this study. The strains collected at the hospital did not have any information that could identify the patient, and only the year of isolation was recorded. All isolates were identified as S. putrefaciens with the automated Microscan WalkAway system (Beckman Coulter, Brea, CA, USA) and as S. putrefaciens or *Shewanella* sp. by retest using the MALDI Biotyper with library version 9 (Bruker Daltonics, Bremen, Germany) at the hospital. Additionally, we used a clinical isolate that had been previously reported but not genetically analyzed (14). In total, nine clinical isolates, each detected from a different patient, from hospitals in Japan were analyzed in this study (Table 1).

In addition, a clinical isolate and 13 environmental isolates of *Shewanella* spp., provided by institutions in Japan or purchased from the National Collection of Type Cultures, were included in the analysis (Table 1).

### Whole-genome sequencing analysis and identification of bacterial species.

Draft whole-genome sequencing analysis of the study isolates was performed with Illumina MiSeq (Illumina, Inc., San Diego, CA), except for S. algae JCM 21037 (=ATCC 51192), for which the draft whole-genome sequence data have already been registered (GenBank accession no. JAAXPX000000000.1). Library preparation, sequencing, and *de novo* assembly for MiSeq were performed as previously reported (15).

For the purpose of comparison, we collected the whole-genome sequencing data of *Shewanella* species isolates, including type strains, deposited in GenBank in October 2019. We employed whole-genome sequencing data in which the 16S rRNA gene nucleotide sequence was more than 1,300 bp and that were identified as *Shewanella* sp. by BLAST search. As a result, whole-genome sequencing data of 20 type strains and 74 other isolates were adopted (Table S1).

The draft genome sequence of the study isolates and registered isolates were compared with fastANI (https://github.com/ParBLiSS/FastANI), and isolates with an average nucleotide identity (ANI) value of 95% or more were clustered (16). If a cluster of isolates included a type strain of a specific species, the isolates within the same cluster were designated species of the type strain.

Additionally, long-read nucleotide sequences were obtained using the MinION sequencer (Oxford Nanopore Technologies, Oxford, United Kingdom) for isolates identified as S. algae clade members (S. algae, *S. carassii*, or *S. chilikensis*) to determine the complete whole-genome sequences. DNA extraction, library preparation, sequencing, and *de novo* assembly for MinION were performed as previously published (17).

### Comparison of *bla*_OXA_ and the surrounding genetic environment.

The *bla*_OXA_ genes in the draft genome sequences of the study isolates were identified and compared with *bla*_OXA_ reference sequences using ResFinder (version 4.1). The genetic environments surrounding *bla*_OXA-55-like_ of S. algae clade isolates identified in our analysis were compared with the reference S. algae JCM 21037 (=ATCC 51192) (type strain) using Easyfig (version 2.2.2). Because only one strain each of *S. carassii* and *S. chilikensis* was identified in the study isolates, the draft whole-genome sequencing data registered in GenBank for these species (*S. carassii*, NZ_NGVS00000000.1; *S. chilikensis*, NZ_MDKA00000000.1 and NZ_NIJM00000000.1) were also included in the analysis.

### Core genome single-nucleotide-polymorphism-based phylogenetic analysis of S. algae isolates.

Core genome single nucleotide polymorphism (SNP)-based phylogenetic analysis was performed using the complete genome sequence of S. algae TUM17379 (accession no. AP024613.1) as the reference. Core genome SNP analysis was performed as previously described (18).

### Cloning of *bla*_OXA-55-like_ genes.

The *bla*_OXA-55-like_ genes of S. algae isolates were amplified by PCR using Platinum *Taq* DNA polymerase High Fidelity (Invitrogen, Carlsbad, CA, USA) with a *bla*_OXA-55-like_ forward primer incorporating an EcoRI digestion site (5′-GATGCATCGAGAATTCATGAATAAAGGTTTGC-3′) and a *bla*_OXA-55-like_ reverse primer incorporating a BamHI digestion site (5′-ATGGACACAGGATCCTCAAGGCAGCAGCTGTTC-3′). The PCR product was purified by the Wizard SV Gel and PCR Clean-Up system (Promega, Madison, WI) and cloned into pCR4-TOPO using the TOPO TA cloning kit for sequencing (Invitrogen) and One Shot TOP10 chemically competent E. coli (Invitrogen). The accuracy of the nucleotide sequences of the inserts was confirmed by Sanger sequencing using the M13 primer (Invitrogen). Subsequently, pCR4-TOPO carrying *bla*_OXA-55-like_ was digested with EcoRI and BamHI (TaKaRa Bio, Inc.) and ligated to the pHSG298 DNA plasmid (TaKaRa Bio Inc.) pretreated with EcoRI and BamHI. The resulting plasmids were chemically transformed into E. coli DH5α. Transformants carrying pHSG298 harboring *bla*_OXA-55-like_ were selected on agar plates containing 50 μg/ml of kanamycin at 37°C for 24 h, and the presence of *bla*_OXA-55-like_ was confirmed with PCR. This experiment was approved by the Toho University Safety Committee for Recombinant DNA Experiment (approval no. 21-52-458).

### Antimicrobial susceptibility testing.

Antimicrobial susceptibility testing was performed for the S. algae clade isolates and E. coli DH5α isolates carrying *bla*_OXA-55-like_ cloned from S. algae isolates by the broth microdilution method using BBL Mueller-Hinton II broth, which was cation adjusted (Becton Dickinson and Co., USA) according to the Clinical and Laboratory Standards Institute (CLSI) guidelines (19). The following antimicrobial agents were used for antibiotic susceptibility testing: ampicillin, piperacillin, cefazolin, cefotaxime, ceftazidime (Sigma-Aldrich, St. Louis, MO, USA), aztreonam (Tokyo Chemical Industry Co., Ltd., Tokyo, Japan), imipenem (Banyu Pharmaceutical, Tokyo, Japan), clavulanic acid, and meropenem (Wako Pure Chemical Industry, Ltd., Tokyo, Japan). E. coli ATCC 25922 and Pseudomonas aeruginosa ATCC 27853 were used as quality control strains. The results were interpreted according to CLSI guidelines (20).

### Quantitative RT-PCR for *bla*_OXA-55-like_.

Quantitative reverse transcription-PCR (RT-PCR) was performed for S. algae clade isolates to compare *bla*_OXA-55-like_ transcription levels. The isolates were grown in LB broth (Becton Dickinson and Co.) for 24 h at 37°C with shaking at 160 rpm and harvested at an optical density at 600 nm (OD_600_) of 1.0. The RNA was extracted using the RNeasy minikit (Qiagen, Hilden, Germany), then used to generate cDNA with PrimeScript RT master mix (TaKaRa Bio Inc.). Quantitative PCR was performed using SYBR green PCR master mix (Applied Biosystems, Foster City, CA) with the primer pair *bla*_OXA-55-like__Forward_Primer (5′-GTTGGTTGGAGTTGGACGAC-3′) and *bla*_OXA-55-like__Reverse_Primer (5′-TGCTTGAGCACCTGTTTCAC-3′) on the Applied Biosystems 7500 Fast system (Applied Biosystems). Amplification of the *rpoB* gene was simultaneously performed with the *rpo*B_Forward_Primer (5′-TTTGATCCCATTCCTTGAGC-3′) and *rpo*B_Reverse_Primer (5′-CCACCAGAGGCTTCTCTGAC-3′). The *bla*_OXA-55-like_ transcription levels of the isolates were compared using the threshold cycle (ΔΔ*C_T_*) method (21). The amplification efficiency of the quantitative PCR for *rpoB* and *bla*_OXA-55-like_ was verified with 10-fold serially diluted TUM17384 total RNA ranging from 10^0^ ng to 10^−5 ^ng per assay, which demonstrated that the ratio of amplification efficiency of *bla*_OXA-55-like_ to *rpoB* was 0.98.

### β-Lactamase activity assay.

β-Lactamase activity in the S. algae clade isolates was evaluated. First, the isolate of interest was inoculated into LB broth (Becton Dickinson and Co.) and incubated for 24 h with shaking at 160 rpm and 37°C. After incubation, 10 ml of the culture medium was centrifuged at 3,500 × *g* for 15 min at 4°C. After the supernatant was discarded, the pellet was washed with 500 μl of phosphate-buffered saline (PBS) (pH 7.0) and recentrifuged at 13,000 × *g* for 1 min at 4°C. After resuspending the pellet in 500 μl of PBS, the crude enzyme solution was prepared by sonication and subsequent centrifugation at 13,000 × *g* for 30 min at 4°C. The protein concentration was measured by the Bradford method using bovine serum albumin (BSA) (Bio-Rad Laboratories, Inc., Hercules, CA) as a standard. The change in absorbance over time caused by the hydrolysis of β-lactam by β-lactamase was measured using a Shimadzu UV-2500 spectrophotometer (Shimadzu, Kyoto, Japan). The β-lactams used as the substrates for β-lactamase were adjusted to a final concentration of 100 μM in PBS. All reactions were performed in a Bandpass 10-mm cuvette with a total volume of 5 μl of enzyme added to 500 μl of substrate solution at 30°C.

### Accession number(s).

The draft whole-genome sequencing data were deposited in GenBank under accession no. BPET00000000.1, BPEU00000000.1, BPEV00000000.1, BPEW00000000.1, BPEX00000000.1, BPEY00000000.1, BPEZ00000000.1, BPFB00000000.1, BPFC00000000.1, BPFD00000000.1, BPFE00000000.1, and BPFF00000000.1. The complete whole-genome sequence data were deposited as GenBank accession no. AP024609 to AP024618.
